# Documentation and communication of nutritional care for elderly hospitalized patients: perspectives of nurses and undergraduate nurses in hospitals and nursing homes

**DOI:** 10.1186/s12912-016-0193-z

**Published:** 2016-12-01

**Authors:** Kristin Halvorsen, Helene Kjøllesdal Eide, Kjersti Sortland, Kari Almendingen

**Affiliations:** 1 Department of Nursing and Health Promotion, Faculty of Health Sciences, Oslo and Akershus University College of Applied Sciences, Oslo, Norway; 2 Division of Medicine, Akershus University Hospital and Institute of Clinical Medicine, University of Oslo, Oslo, Norway

**Keywords:** Older patients, Nutrition and metabolism, Nursing, Documentation, Qualitative research

## Abstract

**Background:**

Nutritional care is a basic human right for all people. Nevertheless, undernourishment is known to be a frequent and serious health care problem among elderly hospitalized patients in Western Europe. Nutritional documentation contributes to ensuring proper nutritional treatment and care. Only a few studies have explored how nurses document nutritional care in hospitals, and between hospitals and nursing homes. Available research suggests that documentation practices are unsatisfactory. The aim of this study was to explore how nurses document nutritional treatment and care for elderly patients in hospitals and how nurses and undergraduate nurses communicate information about patients’ nutritional status when elderly patients are transferred between hospital and nursing homes.

**Methods:**

A qualitative study was conducted using a phenomenological-hermeneutic approach. Data was collected in focus group interviews with 16 nurses in one large university hospital, and 11 nurses and 16 undergraduate nurses in five nursing homes associated with the university hospital. Participants from the university hospital represented a total of seven surgical and medical wards, all of which transferred patients to the associated nursing homes. The catchment area of the hospital and the nursing homes represented approximately 10% of the Norwegian population in heterogenic urban and rural municipalities. Data were coded and analysed thematically within the three contexts: self-understanding, critical common sense, and theoretical understanding.

**Results:**

The results were summarized under three main themes 1) inadequate documentation of nutritional status on hospital admission, 2) inadequate and unsystematic documentation of nutritional information during hospital stay, 3) limited communication of nutritional information between hospital and nursing homes. The three main themes included seven sub-themes, which reflected the lack of nutritional screening and unsystematic documentation on admission and during hospital stay. Further the sub-themes elucidated poor exchange of information between hospital and nursing homes regarding the nutritional status of patients.

**Conclusion:**

Overall, the documentation of nutritional treatment and care for elderly patients was inadequate in the hospital and between health care settings. Inappropriate documentation can create a negative nutritional spiral that leads to increased risk of severe health related complications for elderly patients. Moreover, it hinders nutritional follow-up across health care settings.

**Electronic supplementary material:**

The online version of this article (doi:10.1186/s12912-016-0193-z) contains supplementary material, which is available to authorized users.

## Background

Nutritional care is a basic human right, as stated in Article 25 of Universal Declaration of Human Rights [1]. Nevertheless, undernourishment has been identified as a frequent and serious health care problem among elderly hospitalized patients in Norway and Western Europe in general [2–9]. In a previous study, we found that 45% of elderly hospitalized patients in one large Norwegian university hospital were at nutritional risk [9]. Furthermore, when investigating nutritional care practices, we uncovered that only 1.2% of the elderly hospitalized patients had been screened for nutritional risk using a standardized screening tool [10]. Undernourishment in elderly patients increases the risk of disease-related complications, morbidity, and mortality. It lengthens hospital stay and expands health care costs. Furthermore, it is associated with physical and psychological burdens and reduces quality of life [3, 5, 7, 11–13]. Many elderly patients are already undernourished upon hospital admission, and nutritional status often deteriorates during hospital stay [2, 4, 11, 13–15]. Additionally, elderly patients in general tend to move between different health care settings more often than the younger patient population [16].

In 2009, the Norwegian Directorate of Health published national professional guidelines for the prevention and treatment of undernutrition [15]. These guidelines are in line with the European Society for Clinical Nutrition and Metabolism (ESPEN) guidelines [17] and recommendations for nutritional treatment and care in European health care [18]. Two recommendations highlighted in the Norwegian guidelines specify that nutritional status and treatment have to be documented in patients’ medical records and communicated when patients are transferred between health care settings, for example, between hospital and nursing homes [15].

Appropriate documentation and communication of patients’ needs are regulated by Norwegian health care legislation [19]. A patient’s medical record is a working tool that health care professionals can use to provide safe and adequate patient care. Documented information must be structured and systematized to secure the continuity of treatment and care [19–21]. In order to follow up patients, health care professionals also have an obligation to document and communicate relevant information on patients’ health status between health care settings [19, 20]. Additionally, health care professionals in hospitals are obligated to advise and guide the municipal health care services about patients’ needs [20, 21]. Research has shown, however, that documentation of basic health care needs [22], as well as communication of these needs across health care settings, is generally poor [23–27].

In hospitals, nurses play an important role in following up patients’ nutritional treatment and care [4, 11, 22, 28–30]. To our knowledge, very few studies have explored how nurses in hospital document nutritional care. Additionally, in the studies that have been done, the documentation of nutritional care was found to be unsatisfactory and consisted mainly of information on different eating abilities or disabilities [30, 31]. A limited number of studies have explored how nurses in particular communicate information about the nutritional status of elderly patients between health care settings [24–27]. Research has, however, shown that there are barriers related to inadequate information transfer between health care settings [27, 32]. Barriers that have been highlighted include short hospital stay, resource demands, and discrepancies in nutritional knowledge and skills among health professionals [27, 32].

As only a limited amount of research has focused on documentation practices in nutritional care of elderly hospitalized patients, this study aims to explore how nurses document nutritional treatment and care of elderly hospitalized patients. Additionally, the paper aims to elucidate how nurses and undergraduate nurses communicate nutritional information about elderly patients when those patients are transferred between hospital and nursing homes.

## Methods

This study is part of a larger research project that was carried out using a mixed method approach [33], including one cross-sectional study [9, 10] and two qualitative focus-group studies [32]. In the cross-sectional study, we screened 453 patients for nutritional risk and undernutrition [9] and investigated nutritional care practices in one large university hospital [10]. In the qualitative studies, we elucidated barriers for nutritional care [32] and explored nutritional documentation practices in hospital and nursing homes (this paper).

In the latter qualitative focus group study we used an explorative and descriptive design with a phenomenological-hermeneutical approach. Phenomenology allows us to capture immediate experience-based knowledge, while hermeneutics helps us get at what is beyond immediate understanding and acknowledges the role of pre-understanding in the interpretation of the findings [34, 35].

Focus groups are particularly valuable when the aim is to learn more about people’s experiences, attitudes, and viewpoints in a context where different people interact [36–40], which is the case in this study.

### Setting, recruitment, and sample

The setting for this study was one university hospital in Norway and five nursing homes associated with the university hospital. The hospital is large and provides health care services for approximately 10% of the Norwegian population, living in urban and rural municipalities and is responsible for a heterogeneous population.

We sampled the participants using a purposive sampling procedure. In the hospital, the leading nurse responsible for each of the wards invited the nurses to participate in the focus group interviews, while in the nursing homes, the nursing home manager, the leading nurse on the ward, or a research and development nurse invited the nurses and undergraduate nurses to participate. The researchers received contact information for possible interviewees, who got a formal invitation with information about the study and a consent form.

In the hospital, all the participants were registered nurses (RN), working in seven surgical and medical wards (Table 1). We wanted to talk to the health care professionals who had the daily responsibility for nutritional care of the patients. In total, four focus group interviews were conducted. Three groups consisted of four to six participants each, while the last group only had two participants.

**Table 1:** Characteristics of the participants

Nurses, hospital (*N =* 16)		Nurses and undergraduate nurses, nursing home (*N =* 27)	
Gender, N		Gender, N	
Female	15	Female	25
Male	1	Male	2
Age, years		Age, years	
Mean	29.3	Mean	44.6
Range	23-47	Range	23-64
Type of ward, N		Health profession, N	
Orthopedic	3	Nurse	11
Upper gastro	2	Undergraduate nurses	16
Lung	4		
Cardiology	3		
Hematology/infection	1		
Neurology/endocrinology	2		
Neurology/stroke	1		
Experience as nurse, years		Type of unit, N	
Mean	5.7	Long-term	14
Range	1-21	Short-term	8
		Long-term + short-term	4
		Special care (dementia)	1
Experience with elderly patients, N		Professional work experience, years	
Some	5	Mean	17.5
Much	11	Range	0.25-40

In the nursing homes, the participants were registered nurses (RN) and undergraduate nurses, working primarily in long- and/or short-term somatic wards. In Norwegian nursing homes, undergraduate nurses are given major responsibilities in both planning and implementing nutritional care, and for that reason were invited to participate. Five focus groups, each consisting of five to six participants, were conducted, one at each nursing home (Table 1).

In both the hospital and the nursing homes, all focus groups consisted of participants representing different wards to give broad perspectives and to enrich the discussions. To participate, nurses had to have worked in a 50% position or more in the same hospital or nursing home ward in the three months preceding the study.

### Data collection

A pilot study was conducted in March 2012, which consisted of two focus groups, one with nurses in a hospital setting and one with nurses and undergraduate nurses in a nursing home setting. In the pilot interviews we determined that the participants seemed to talk more about what they ought to do and what they thought would be the best practice, rather than what they actually did. For that reason, following discussion among the co-authors, the interview guides were adjusted to ask more directly about participants’ actual practical work experiences of nutritional care, including documentation practices.

The focus group interviews were conducted in spring and fall 2012; they were led by a moderator (second author) and an assistant (first author) [34, 40] to capture the complexity of the group settings [35, 41]. The moderator is a clinical dietitian and PhD student with experience as a nurse assistant in home care services. The assistant is an experienced intensive care nurse with an MNSc and a PhD in medical ethics.

Each focus group interview lasted around 90 min. During the interviews, we used the revised interview guide. Key topics concentrated on experiences related to how the documentation of nutritional status and treatment were carried out in the hospital. Additionally, we focused on how nutritional information was communicated between the hospital and the associated nursing homes.

The focus groups met in quiet rooms at the hospital and in the nursing homes and we served light drinks and refreshments. The goal was to promote an open and trusting atmosphere that would give unanticipated statements and personal experiences the chance to emerge. We continued to perform focus group interviews until data were saturated and no new data emerged during the discussions. The interviews were audiotaped and transcribed verbatim by the moderator shortly after each focus group. Transcripts and recordings were checked and cross checked to avoid transcription errors. During the interviews, the assistant also made field notes and was responsible for validating answers by summing up the discussions with input and corrections from the participants, as recommended by Kvale and Brinkmann [35].

### Data analysis

The data generated were analysed in three interpretative contexts as proposed by Kvale and Brinkmann [34]. These contexts are self-understanding, critical common sense understanding, and theoretical understanding [34]. In the analysis, we sought a balance that recognised the interplay between individual and group statements [38–41]. In the self-understanding context, we tried to capture what the subjects understood as the meaning of their statements. To do this, we identified meaning units in each interview. In the context of common sense understanding, data were coded inductively, although still guided by the research questions [34, 40]. The first cycle of coding the transcript was done by the first author. Both the first and second authors read all transcripts and the field notes several times to get a sense of the whole before agreeing on the main codes. Based on the main codes, we coded the data into sub-codes. Main codes and sub-codes were organized into main themes and sub-themes [34, 40]. In the context of theoretical understanding, relevant white papers, theory, and research on nutritional care were applied to interpret the findings in a broader perspective.

### Rigour

With small samples, as in qualitative research, heterogeneity may make it difficult to transfer the results to other settings [34]. This study, however, included 43 participants from seven different hospital wards and five nursing homes to obtain sufficient data to capture the range of variations in the nurses’ experiences in documenting and communicating nutritional care. The nurses and undergraduate nurses also had different background variables, such as work experience, age, gender and ward characteristics. Variances in background variables may facilitate broader descriptions of experiences and thereby better capture core elements of the participants’ experiences of documentation and communication of nutritional care for elderly hospitalized patients. A sample of quotes from the participants has been used in presenting the results to paint a broader picture of the findings. Additionally, to identify targeted participants for the study, we discussed the question of which health professionals were most appropriate to include in the study with health professionals from the clinics, the research and development department (R&D) and the clinical dietitians at the university hospital.

During the data collection and analysis we acknowledged, reflected on, and discussed our pre-understandings. We experienced that the open atmosphere in the focus groups created a comfortable situation were the participants could speak freely, using their own words, sharing and discussing their experiences. The findings were also validated by the authors reading the transcripts separately and then discussing their understanding throughout the analytical process.

### Research ethics

The study was approved by the university hospital’s internal privacy commission and by the management of the hospital and the nursing homes. All participants gave their written voluntary informed consent.

## Results

The results are organized into three main themes, with sub-themes that complete and elaborate the main themes. All themes have their foundation in the research issues of the study. The themes are systemized according to nutritional documentation at hospital admission and during hospital stay and documentation and communication of nutritional information upon transfer between hospital and nursing home. The themes and sub-themes are shown in Table 2.

**Table 2:** Overview of the main themes and sub-themes of the results

Main themes	Sub-themes
Insufficient documentation of nutritional status on hospital admission	No documentation of nutritional risk using validated screening tools Unsystematic and lacking documentation of nutritional information by hospital admission
Inadequate and unsystematic documentation of nutritional information during hospital stay	Documentation of eating habits and special meals Unstructured documentation of nutritional information in the patient’s medical record Limited attention on nutritional documentation
Limited communication of nutritional information between hospital and nursing homes	Limited nutritional information from the nursing homes to the hospital Limited nutritional information from the hospital to the nursing homes

### Insufficient documentation of nutritional status on hospital admission

Our findings showed that documentation of nutritional information on patient admission was experienced as insufficient and arbitrary. According to the participants, elderly patients were hardly ever screened for nutritional risk. Other documentation of nutritional information, such as weight, appetite and nutritional needs occurred randomly, according to the nurses, and was perceived as incomplete.

#### No documentation of nutritional risk using validated screening tools

In spite of the guideline from the Norwegian Directorate of Health [14] to perform and document nutritional risk screening for all patients on hospital admission, the participants reported that this was not practiced when elderly patients were admitted to hospital. ‘We don’t have any screening tool for nutritional risk that we use’, one participant explained (participant 13).

Only a few of the nurses reported having heard about nutritional risk screening tools and/or the national professional guidelines on prevention and treatment of undernutrition.

We do not use those [screening tools] in practice and we have not heard about the guidelines (participant 9). Yes, I have heard about them [screening tools] (participant 8).

In response to a question about how they then could recognize and document undernourishment, or make a nutritional care plan, when patients were not screened for nutritional risk and/or undernourishment their answers were quite vague:

Observation (participant 11). Clinical judgments (participant 12). We probably recognize them [the undernourished] when they are admitted, but we do not always document it (participant 11).

#### Unsystematic and lacking documentation of nutritional information on hospital admission

According to the participants, some nutritional information was documented for elderly patients on admission to hospital, such as data on weight, likes and dislikes regarding food, and diet. However, they expressed that the information gathered about nutritional status was arbitrary. Most of the time, as the nurses experienced it, the documentation provided few directions for nutritional treatment or prevention undernourishment during hospital stay.

It may happen that the physicians write that the patient seems undernourished, but I do not feel that this gives any directions for further treatment and it might as well not be documented in the incoming medical record either (participant 1). Most of the time they document ‘in normal condition’ (participant 3)

Patient weight was one parameter that seemed to be occasionally documented, for example, if the patient suffered from a diagnosis that implied weight loss. However, according to the participants, most of the time patients were not actually weighed but were asked to estimate their weight instead. In other words, it was the anticipated weight that was documented and not the actual weight.

It is quite often the patient is asked about their current weight, in the emergency room, when admitted. We rarely see other methods for documentation on undernutrition. However, if it is very obvious, weight loss may be documented (participant 16)

Documentation using the international diagnostic codes for undernutrition in the ICD-10 coding system occurred very rarely, and very few of the participants said they had ever heard that such coding was possible.

I don’t think I have seen it ever in our ward (participant 13).

I have never seen it in the discharge summaries. So I do not know if they [the physicians] use the diagnosis code and document it (participant 14).

To summarize, these findings indicate a serious lack of documentation of relevant nutritional information at hospital admission.

### Inadequate and unsystematic documentation of nutritional information during hospital stay

The participants, as nurses, experienced being the main contributors in documenting nutritional information for elderly patients. However, the findings suggest that the nutritional information was not systematically and adequately documented. The documentation seemed to be rather superficial and lacked in-depth information on the patients’ needs in terms of nutritional treatment and care.

#### Documentation of eating habits and special meals

According to the participants, documentation of nutritional information was often related to food intake, appetite, and physical abilities influencing eating. However, they realized that this data provided little information when it came to the actual food eaten and its nutritional value. The nutritional information was perceived as vague and imprecise.

As I said, we do not have any concrete amount that we have measured before we give the food to the patient. Then we often document: ‘the patient ate a small portion’, and what is a small portion? Is it two teaspoons or ….. (participant 11)? Yes, what is a small portion (participant 14)?

The participants reported that nutritional treatment and/or a nutritional treatment plan was hardly ever documented. When nutritional treatment was documented and a treatment plan was set, it mainly happened if the patient was obviously very thin and undernourished. The nutritional treatment most often documented was supplement drinks or energy- and nutrient-enriched meals. Additionally, when patients seemed very thin and undernourished they were occasionally referred to a clinical dietitian. In those situations, a nutritional treatment plan and follow-up were usually documented in the medical record. Decisions about nutritional supplements or whether to refer the patient to a clinical dietitian were usually based on the nurse’s visual impression that the patient was undernourished, and maybe blood tests, but not on nutritional screening. As one of the nurses said … ‘if they look very thin…’ (participant 4).

I may ask the physician to write it in the medical record. If he documents the recommendation of supplement drinks, he may write ‘twice a day or four a day’. You have to document in the medical record that it has been given, as you do with any other medication. It is seen as a kind of treatment. Sometimes they even decide what kind of supplement drink they want the patient to have (participant 16).

Interestingly, according to the participants, documentation on fluid restrictions and balance seemed to be better.

We document fluid restrictions, regarding patients’ with heart failure, admitted because they need to be dewatered (participant 16).

#### Unstructured documentation on nutritional information in the patient’s medical record

Participants reported a lack of structure in the documentation of nutritional information in patients’ medical records. They reported that the documentation system was complex and difficult to navigate, in part because nutritional information was often documented in several different places in the medical record, and difficult to find.

Often you have to scroll far back to find the weight of the patient (participant 9). It [nutritional information] disappears in the electronic medical record (participant 10).

Another participant described ‘the navigation problem’ in the medical record like this:

Often, I triple document because I am afraid that the others do not read the treatment plan. Therefore I write in the report as well, in the mark area, and then I have written it three different times, in three different places (participant 16).

Participants also reported lacking competence and therefore having difficulties documenting the nutritional status of elderly patients. They struggled with knowing what they ought to document, as well as with where to document it. For example, one participant said, ‘I do not think it is easy to document it either, what I consider their nutritional status to be’ (participant 1).

#### Limited attention to nutritional documentation

The participants experienced limited attention to nutritional documentation and related this to short hospital stays and their hectic working days. The nurses perceived that the focus during hospitalization was on the patients’ medical diagnosis and associated medical treatment rather than on nutritional care. Such care seemed to be a ‘missing link’ in the understanding of the relationship between the patients’ medical condition and undernutrition.

Our focus is medical treatment and we are so specialized (participant 12). It is a lot of pressure to discharge the patient and then nutritional care comes far behind (participant14).

The participants also experienced that the physicians were often not interested in documenting nutritional information. The nurses also felt that being met with an attitude that suggested they were writing ‘unnecessary’ information made them hesitant to document nutritional information.

The other day we had a physician who became irritated and wanted to exclude the documentation from the nurses from ‘his list’. He was not interested in knowing what he [the patient] had eaten for dinner (participant 16).

### Limited communication of nutritional information between hospital and nursing homes

Communication of relevant nutritional information for elderly patients was a two-way task. Interestingly, both the hospital and nursing home participants considered it not good enough. As the findings show, they also had differing perceptions of the quality of their own information and they were displeased with each other’s communication.

#### Limited nutritional information from the nursing homes to the hospital

According to the participants in the hospital, they very rarely received information about the nutritional status of elderly patients transferred from the associated nursing homes.

No, very rarely do we receive nutritional information, actually, from the nursing home. If there is any, it is a note from the admitting physician (participant 16).

However, whether or not nutritional information was obtained depended on the actual nursing home. However, the participants from the hospital felt in general that the nutritional information they received from the nursing homes was rather limited.

It depends on the nursing home, if they send documentation on nutritional information. If they send anything, it is a list where height and weight sometimes is added (participant 12). I think I seen that twice in a year (participant 11).

Some of the participants reported that even important information regarding physical ability to eat was not communicated properly from the nursing homes, such as ‘if they eat pureed food, or…’ (participant 9). Participants from the hospitals reported that they regularly experienced that necessary information, such as if patients suffered from paralysis that affected their ability to swallow or chew, was not documented in the transfer papers or the patients’ medical record.

However, the participants from the nursing homes claimed that they gave some nutritional information to the hospital when a patient’s condition required hospitalization.

If the elderly patient has an acute hospital admission we usually write a nursing summary. If the patient is undernourished we communicate ‘bad appetite’, ‘little food intake’ (participant 39).

#### Limited nutritional information from the hospital to the nursing homes

The participants in the hospital reported that they gave sufficient nutritional information to the associated nursing homes when elderly patients were transferred from the hospital.

I think we are quite good actually, in communicating nutritional information when the patients are discharged from the hospital and transferred to a nursing home. We write a proper nursing summary that includes nutritional status. It is of poorer quality when the patients are admitted from nursing homes to the hospitals (participant 8).

When talking to the participants in the nursing homes, they, on the other hand, reported that the nutritional information they received from the hospital was of poor quality and was given low priority.

The communication with the hospital is in general extremely bad. Regarding nutritional information, I believe it is coming so far down the ladder (participant 43), I have hardly never seen that weight is communicated [in the nursing summaries] (participant 39).

The consensus from all the nursing home focus groups was that information from the hospital about patient nutritional status was scarce and, in most cases, did not include nutritional information of value for the nutritional treatment and care of the transferred patients Additional file 1. Additional quotes are included in Additional file 2.

When we considered experiences from both the hospital and the nursing homes related to the communication of nutritional information, it seemed like the health care professionals we interviewed had no clear directions as to what nutritional information to document, and where and how to communicate sufficient and necessary nutritional information to each other. Both the hospital and the nursing homes were displeased with the nutritional information they received. Surprisingly, they were both quite pleased with themselves regarding the information they gave the other. The findings did, however, indicate that communication about elderly patients’ nutritional status between hospital and nursing homes has to be improved in terms of quality and routines.

## Discussion

Overall, we found that neither the documentation of nutritional treatment and care for elderly hospitalized patients nor the transfer of nutritional information between health care settings was sufficient. The obligation to document patients’ needs for treatment and care is enshrined in health legislation, in several white papers, and in international human rights [1, 15, 16, 19, 21], but our findings indicate that this is not the case for nutritional treatment and care. Based on our findings it is imperative to ask: Why are nutritional documentation and communication so inadequate, and what are the consequences of this inadequacy?

### Possible reasons for inadequacy of nutritional documentation

According to our findings, the context of the acute care setting may be one of the reasons for neglecting nutritional care and an excuse for the inadequacy of nutritional documentation. The average length of stay for the elderly patients in the participating hospital wards was two to four days. The treatment and care focus was mainly on the admitting diagnosis and, as a result, nutritional care was not given sufficient attention and guidelines, such as weighing the patient on admission, were not followed [15]. Inadequate focus on nutritional care due to short hospital stay is also documented in other comparable studies [28, 29, 42]. It is worrying that nurses do not seem to properly take into consideration the correspondence between patients’ medical situation and their nutritional status, and the serious risk of increased suffering for patients posed by undernutrition [3–7, 11–13].

The lack of documentation and communication of nutritional information could partly be explained by what the participants described as unsystematic and unstructured documentation practices. There were no clear routines for nutritional risk screening, nutritional treatment, or communication of nutritional information between the hospital and the associated nursing homes, although this is recommended in the Norwegian nutritional guidelines [14]. Research has shown that nutritional screening has the potential to reduce undernourishment in elderly hospitalized patients; however, the findings point in slightly different directions [5–7, 43–46]. Nutritional risk screening practices vary among Norwegian university hospitals [7, 10]. While some have established routines for nutritional risk screening and nutritional guidelines [7, 8], others do not [10, 33].

A patient’s medical record is the most important tool to ensure that they receive the right treatment, care, and continuity of treatment [22–24]. The participants in our study expressed that nutritional documentation in general was difficult to find, as it often was located in different places in the patients’ electronic medical records. The term ‘triple documentation’ occurred several places in the data material to describe the difficulty of figuring out where to document nutritional care. Care providers are required by law to document a patient’s need for treatment and care [19, 20], but according to our findings, this standard is in jeopardy in clinical practice for elderly patients with regard to nutritional care.

Adequate documentation of nutritional status, treatment, and care presupposes nutritional competence, which has been shown to be inadequate in several studies [5, 28, 29, 32]. Nurses mainly document information about patients’ ability or inability to eat, and documentation of actual nutritional status is lacking [28, 29]. Our data showed similar findings, with most documented nutritional information being about food, for example, appetite, likes, dislikes, diets, and food intake. Although these descriptions are relevant to nutritional care, the documentation for individual and targeted nutritional care was not satisfactory.

### Difficulties in following up nutritional needs

When nutritional status is not documented properly it becomes impossible to follow up nutritional needs for elderly patients in hospitals or give proper nutritional information when older patients are transferred back and forth between hospitals and nursing homes. In Norwegian health care, the focus has been on making transfer between hospital settings more seamless [21]. However, seamless transfer between health care settings presupposes accurate documentation of current needs and adequate systems for how and where to document nutritional information, as well as guidelines for what information should be documented. There is need for a common language regarding nutritional treatment and care between the sectors. Nutritional information has to be given increased attention when patients are moving between health care settings [15].

### Negative nutritional spiral

Undernutrition can very easily become a continuous negative nutritional spiral. Undernutrition prolongs hospital stay, increases the risk of complications, mortality and morbidity, raises health care costs, and leads to depressive symptoms and decreased quality of life [2–6, 13, 15]. Better awareness of nutritional status and treatment has great potential to improve elderly patients’ health considerably. Nevertheless, research has uncovered that nutritional status often deteriorates during hospital stay. Inadequate attention to nutrition, and deficiencies in nutritional documentation will continue the negative nutritional spiral [15], with serious consequences for the elderly patients. Health professionals must assume their interdisciplinary responsibility when it comes to caring for elderly patients’ nutritional needs, including in-hospital documentation and communication of nutritional information across health care settings. Interdisciplinary responsibility involves a clarification of roles in nutritional treatment and care. One reason for the lack of focus on nutritional documentation might be confusion and uncertainty regarding responsibility. Considering the involved parties’ professional background, the actual competence of the professionals caring for these patients appears to be good. However, it seems like they are ‘not playing in the same orchestra’, which may involve a serious risk for the patients. Roles and responsibilities must be clarified in such a way that acknowledges the key position of nurses who are close to the patients around the clock.

### Strengths and limitations

The results from qualitative studies are not generalizable [33]. However, our results may be transferable to other similar contexts since the hospital and associated nursing homes in this study provide healthcare services for a large catchment area and the participants represent valuable variance.

Our research only exposes the nurses and undergraduate nurses’ experiences. We did not speak with physicians, nor did we read patients’ medical records, discharge summaries from nurses and physicians, or incoming summaries from nursing home transfers. Including interviews with other responsible health personnel and analysis of written documentation would have enriched the study, but it also would have shifted its focus.

The researchers’ preconceptions have an influence on the research process [33, 39, 47]. To ensure a rigorous presentation of the results, all authors were involved in the analysis. Reliability and validity were additionally provided by performing the research according to the consolidating criteria of the COREQ 32-item checklist [48] (Additional file 3).

#### Implications for nursing practice, education and research

Undernourishment presents a large challenge in caring for elderly hospitalized patients in Norway. Nurses and undergraduate nurses play a major role in ensuring that elderly patients in the Norwegian health care system meet their nutritional requirements. Influencing their approach and attitude to nutritional care may therefore yield clinical benefits. This study elucidates how imperative it is to have a system in place that makes it possible for nurses to identify and document nutritional risk and initiate appropriate nutritional treatment and care. Documented treatment plans that include descriptions of individual nutritional care are necessary to safeguard elderly patients, secure their quality of life, and control health care costs.

Nursing education has to emphasize the impact of nutritional competence and care in its curriculum and learning outcomes. Research focusing on nutritional care from different perspectives and methodologies is imperative to ensure evidence-based nutritional practices.

## Conclusion

This study highlights that the documentation of nutritional treatment and care was inadequate, both on hospital admission and during hospital stay for elderly hospitalized patients. Moreover, nutritional information was seldom properly communicated when elderly patients were transferred between the hospital and the associated nursing homes. Inappropriate documentation of nutritional information perpetuates a negative nutritional spiral that leads to increased risk of serious health-related complications, longer hospital stays, reduced quality of life, and increased health care costs. Moreover, it hinders nutritional follow up across health care settings.

## Additional files

Additional file 1: Participants in the focus groups. (DOCX 12 kb)
Additional file 2: Quotes on nutritional information between the hospital and nursing homes. (DOCX 13 kb)
Additional file 3: COREQ 32-item checklist. (DOCX 15 kb)

## Abbreviations

ESPEN: European Society for Clinical Nutrition and Metabolism
HKE: Helene Kjøllesdal Eide
KA: Kari Almendingen
KH: Kristin Halvorsen
KS: Kjersti Sortland
MNSc: Master of Nursing Science
PhD: Philosophiae Doctor
R&D: Research & Development
RN: Registered Nurse
