# Supplementary material for: Documentation and communication of nutritional care for elderly hospitalized patients: perspectives of nurses and undergraduate nurses in hospitals and nursing homes
Source: BMC Nurs. 2016 Dec 1;15:70. doi: 10.1186/s12912-016-0193-z (PMC5134106; doi:10.1186/s12912-016-0193-z)
Supplement: Additional file 1: — Participants in the focus groups. (DOCX 12 kb) [file 12912_2016_193_MOESM1_ESM.docx]

Supplementary file 1: Participants in the focus groups

| Number of focus group | Settings | Number of participants |
| --- | --- | --- |
| Focus group 1 | Hospital | 1-6 |
| Focus group 2 | Hospital | 7-10 |
| Focus group 3 | Hospital | 11-14 |
| Focus group 4 | Hospital | 15-16 |
| Focus group 5 | Nursing Home | 17-23 |
| Focus group 6 | Nursing Home | 24-28 |
| Focus group 7 | Nursing Home | 29-35 |
| Focus group 8 | Nursing Home | 35-38 |
| Focus group 9 | Nursing Home | 39-43 |
