# Supplementary material for: Documentation and communication of nutritional care for elderly hospitalized patients: perspectives of nurses and undergraduate nurses in hospitals and nursing homes
Source: BMC Nurs. 2016 Dec 1;15:70. doi: 10.1186/s12912-016-0193-z (PMC5134106; doi:10.1186/s12912-016-0193-z)
Supplement: Additional file 2: — Quotes on nutritional information between the hospital and nursing homes. (DOCX 13 kb) [file 12912_2016_193_MOESM2_ESM.docx]

Supplementary File 2: Quotes on nutritional information between the hospital and nursing homes

| Focus group 5 | I feel there is no focus on nutrition (participant 25). We find out ourselves (participant 24) […]. We do get nursing summaries from the hospital where they write ‘bad appetite etc.’ (participant 26) […]. Sometimes they call us. However, it does not happen often (participant 23). |
| --- | --- |
| Focus group 6 | Sometimes the nursing summary includes undernutrition and that nutritional drinks have been prescribed (participant 28) […]. Nothing much on weight or weight loss (participant 29) […] or what they have eaten […]. And usually when patients have been in hospital they have a bad appetite, so I do not pay much attention to that […]. Of course, they have been in hospital for a reason (participant 30). |
| Focus group 7 | I do not think they are good at describing what they have eaten or prefer to eat when they are transferred from the hospital (participant 36). […]. The documentation mainly says that they have eaten ‘normal food’ (participant 37) |
| Focus group 8 | We mainly get information such as ‘bad appetite’ (participant 39). ‘Eating very little’. Short descriptions of nutritional status (participant 42) |
