# Supplementary material for: Documentation and communication of nutritional care for elderly hospitalized patients: perspectives of nurses and undergraduate nurses in hospitals and nursing homes
Source: BMC Nurs. 2016 Dec 1;15:70. doi: 10.1186/s12912-016-0193-z (PMC5134106; doi:10.1186/s12912-016-0193-z)
Supplement: Additional file 3: — COREQ 32-item checklist. (DOCX 15 kb) [file 12912_2016_193_MOESM3_ESM.docx]

Supplementary file 3: COREQ 32-item checklist

Tong A, Sainsbury P, Craig G. Consolidated criteria for reporting qualitative research (COREQ): a 32-item checklist for interviews and focus groups. *International Journal for Quality in Health Care.* 2007; 19: 349-357*.*

Supplement 2: COREQ 32-item checklist

**Domain 1: Research team and reflexivity**

Personal characteristics of interviewer

1. Interviewer (moderator): Helene Kjøllesdal Eide
2. Credentials: Clinical Dietitian
3. Occupation: PhD student
4. Gender: Female
5. Experience and training: Experience from working in the home care services. Research experience from writing a master’s degree thesis on undernutrition and elderly nursing home residents.

Relationship with participants

1. Relationship established: A relationship to the participants was not established prior to the focus groups.
2. Participant knowledge of the interviewer: Participants were informed of the PhD work and the research goals as well as of the interviewer’s role in the focus group interviews.
3. Interviewer characteristics: Participants were informed of the educational and occupational background of the interviewer.

Personal characteristics of facilitator

1. Facilitator (assistant): Kristin Halvorsen
2. Credentials: RN (ICU), MNSc, PhD
3. Occupation: Associate professor
4. Gender: Female
5. Experience and training: Experienced qualitative researcher within different qualitative approaches.

Relationship with participants

1. Relationship established: The participants were not acquainted with the facilitator prior to the focus group.
2. Participant knowledge of the facilitator: Participants were informed of the role of the facilitator during the interview. They were also informed that the facilitator was the supervisor for the PhD student (interviewer).
3. Facilitator characteristics: Participants were informed of the educational and occupational background of the facilitator.

**Domain 2: Study design**

Theoretical framework

1. Methodological orientation and Theory: Hermeneutic-phenomenological scientific approach.

Participant selection

1. Sampling: Participants, nurses (RN) and undergraduate nurses working in a 50 % or more position were sampled by a *purposive sampling procedure*.
2. Methods of approach: The lead ward nurses or a Research & Development nurse in the wards recruited participants. The second author then distributed written information about the research and a consent form by email.
3. Sample size: Hospital 16 RN; Nursing homes 11 RN and 14 undergraduate nurses.
4. None of the approached participants refused to participate or dropped out.

Setting

1. Setting of data collection: In a quiet room in the hospital and nursing homes.
2. Presence of non-participants: No non-participants were present.
3. Description of sample: RNs sampled from one large university hospital, covering 10 % of the Norwegian population in rural and municipal areas. RNs and undergraduate nurses from five nursing homes associated with the hospital. For a more specific description of sample, see Table 1.

Data collection.

1. Interview guide: An interview guide was used, developed by the first and second authors. All four authors responded on the interview guide. The first and second author conducted two pilot focus group interviews.
2. Repeat interviews: There were no repeat interviews.
3. Audio/visual recording: The interviews were audiotaped and transcribed verbatim.
4. Field notes: The facilitator took notes during the focus group interviews.
5. Duration: Four hospital interviews were conducted between April and June 2012 and five nursing home interviews were conducted between October and December 2012.
6. Data saturation: The researchers agreed that the data were saturated. Nothing new emerged from the data in the last interviews in both the hospital and the nursing homes.
7. Transcripts returned: Transcripts were not returned to the participants. The facilitator made a summary of discussed topics during each interview session for the participants to comment on.

**Domain 3: Analysis and findings**

Data analysis

1. Number of data coders: The first author (facilitator) coded the data manually for this paper and the interviewer and facilitator discussed and agreed upon the codes.
2. Description of coding tree: The coder developed a “working coding tree” used in development of the main- and sub-themes.
3. Derivation of themes: The themes were derived from the codes. The codes and themes are closely connected to the study aims and research questions.
4. Software: Not used.
5. Participant checking: The participants have not yet been given feedback on these findings but will get the published paper.

Reporting

1. Quotations presented: Quotations from 15 different participants are presented to illustrate the themes. The quotations are identified by participant number.
2. Data and findings consistent: Data and findings were discussed thoroughly and several times in the research team to ensure consistency.
3. Clarity of major themes: Major themes are clarified by short explanations and quotations.
4. Clarity of minor themes: Minor themes nuance major themes and are clarified by short explanations and quotations. Diverse cases are made visible.
